# Evaluation of Epigallocatechin-3-Gallate as a Radioprotective Agent During Radiotherapy of Lung Cancer Patients: A 5-Year Survival Analysis of a Phase 2 Study

**DOI:** 10.3389/fonc.2021.686950

**Published:** 2021-06-10

**Authors:** Wanqi Zhu, Yalan Zhao, Shuyu Zhang, Xiaolin Li, Ligang Xing, Hanxi Zhao, Jinming Yu

**Affiliations:** ^1^ Tianjin Medical University Cancer Institute and Hospital, National Clinical Research Center for Cancer, Key Laboratory of Cancer Prevention and Therapy, Tianjin, Tianjin’s Clinical Research Center for Cancer, Tianjin, China; ^2^ Department of Radiation Oncology, Tianjin Medical University, Tianjin, China; ^3^ Department of Radiation Oncology, Shandong Cancer Hospital and Institute, Shandong First Medical University and Shandong Academy of Medical Science, Jinan, China; ^4^ Department of Oncology, The Affiliated Hospital of Southwest Medical University, Luzhou, China; ^5^ Second Affiliated Hospital of Chengdu Medical College, China National Nuclear Corporation 416 Hospital, Chengdu, China

**Keywords:** Epigallocatechin-3-gallate, lung cancer, radiation-induced esophagitis, radioprotective agent, long-term follow-up

## Abstract

**Background:**

Previous analysis of the study (NCT02577393) had demonstrated the application of epigallocatechin-3-gallate (EGCG) could be safe and effective in the prevention and treatment of acute radiation esophagitis in patients with advanced lung cancer. EGCG seemed to improve the response rate of small cell lung cancer (SCLC) to radiotherapy in a subgroup analysis. This research continued to analyze the impact of EGCG application on cancer-radiation efficacy and patient survival.

**Methods:**

All patients with SCLC in the NCT02577393 study were included. Patients were randomized into EGCG group or conventional therapy group as protocol. The primary endpoints of the study were radiation response rate and progression-free survival (PFS). Overall survival (OS) and the efficacy of EGCG in the treatment of esophagitis were assessed as secondary endpoints.

**Results:**

A total of 83 patients with lung cancer in the NCT02577393 study were screened, and all 38 patients with SCLC were eligible for analysis. No significant differences with regard to baseline demographic and clinical characteristics were observed between the two groups. The objective response rate (ORR) was higher than that of conventionally treated patients (84.6 *vs* 50%, *P* = 0.045), while the median PFS and OS were not significantly prolonged. At data cut-off (1 January 2021), 5-year PFS was 33% with EGCG *versus* 9.3% with conventional treatment, and 5-year OS was 30.3% *versus* 33.3%, respectively. The mean adjusted esophagitis index and pain index of patients with EGCG application were lower than conventional treatment (5.15 ± 2.75 *vs* 7.17 ± 1.99, *P* = 0.030; 8.62 ± 5.04 *vs* 15.42 ± 5.04, *P* < 0.001).

**Conclusion:**

The study indicates EGCG may alleviate some esophagitis-related indexes in SCLC patients exposed to ionizing radiation without reducing survival. However, this conclusion should be confirmed by further studies with large sample size.

## Background

Acute radiation-induced esophagitis (ARIE) is a typical adverse reaction that occurs in patients with chemo-radiotherapy/radiotherapy, which is more common in lung cancer (1). The incidence of grade 2–3 acute esophagitis caused by CCRT is 20–53.4% in pulmonary carcinoma (2, 3). The most common symptoms in patients with ARIE are odynophagia and dysphagia two or three weeks after radiation (4). With increasing attention to ARIE, new strategies for preventing and mitigating it have become an active research field. Assuredly, severe ARIE is positively correlated with the high-dose radiation per unit volume of the esophageal mucosa (5). Great efforts are being made to overcome its risk through the development of novel radiation technology and treatment targeting related signaling pathways (6).

Epigallocatechin-3-gallate (EGCG) is the main component of tea polyphenols, accounting for an average of 65% in the total tea polyphenols. It has been proved to have a strong protective effect against radiation-induced damages in the normal tissue on the cellular and animal level (7–9). Recently, the anti-irradiation damage activity of EGCG has been preliminarily proved in clinical trials, with our data confirming that the application of plant-derived polyphenol can ameliorate ARIE, radiation mucositis, and radiation dermatitis (10–16). The safety and effectiveness of EGCG make it one of the promising candidates for radioprotection. No tumor-damaged repair is also an important consideration for developing the agent, just like ensuring efficacy and acceptable toxicity. Interesting, EGCG seems to have a certain radio-enhancing effect on SCLC during radiotherapy in clinical practice. Therefore, the radiation efficacy and survival follow-up of patients with SCLC in the published trial (NCT02577393) were analyzed for verifying the overall role of EGCG in tumor radiotherapy.

## Methods

### Study Population and Study Design

NCT02577393 study as a three-arm, controlled, randomized, prospective study was conducted to explore the preventive and therapeutic action of EGCG against ARIE in patients with the combination of chemoradiotherapy. The protocol was available at *Oncology and Radiotherapy* online (10). EGCG was purchased from NINGBO HEP Biotech Co., Ltd and dissolved in 0.9% saline solution to make the concentration up to 440 umol/L with reference to the results of phase I study (16).

**Figure 1:**
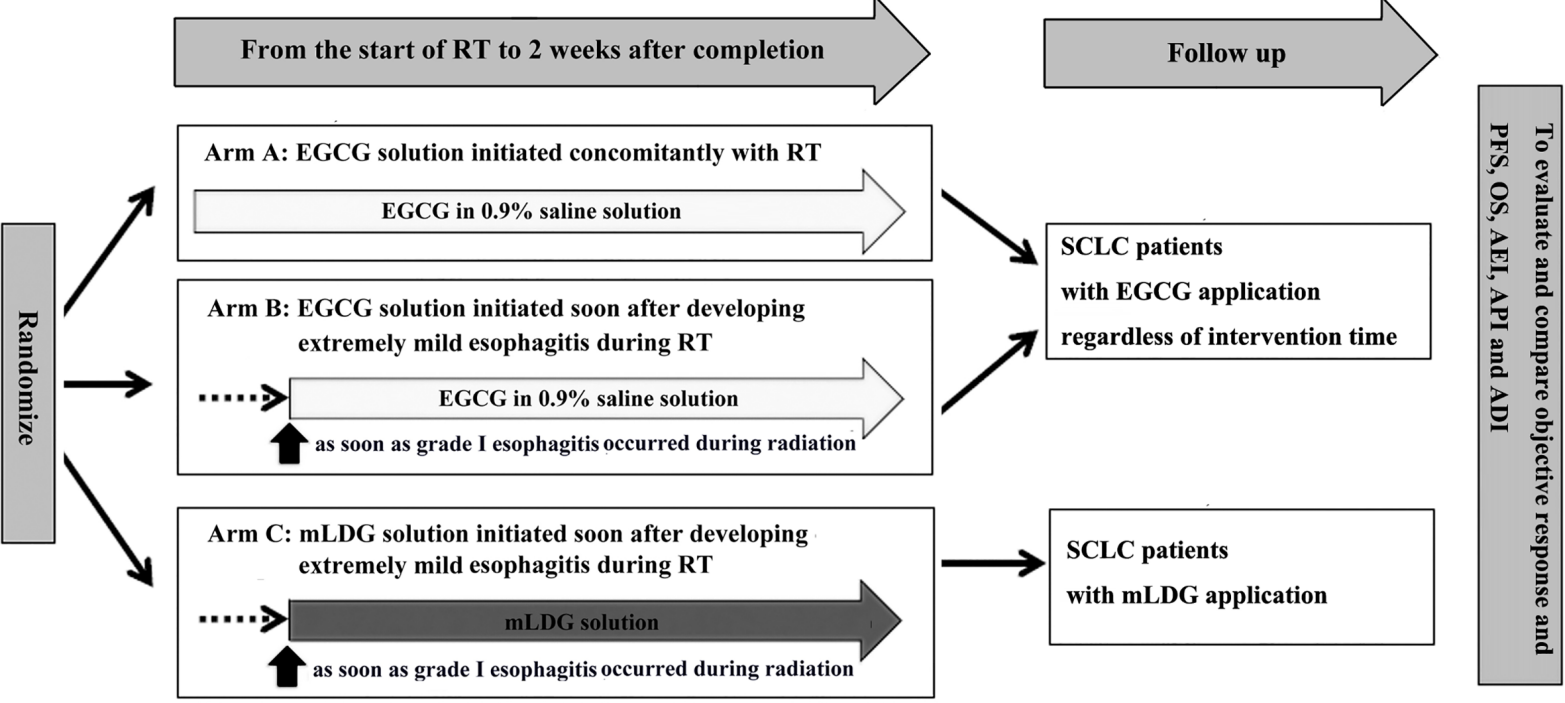
An overview of the study design. EGCG, Epigallocatechin-3-gallate; PFS, progression-free survival; OS, overall survival; AEI, adjusted esophagitis index; API, adjusted pain index; ADI, adjusted dysphagia index.

The analysis described here included patients with SCLC who received EGCG or conventional treatment in the NCT02577393 study. Patients who slowly swallowed EGCG solution with 10 ml three times daily, whether at the beginning of radiation or at the appearance of grade I esophagitis, were included in the EGCG group. The patients in the conventional treatment group were those who were treated with a solution containing 0.16 mg/ml lidocaine, 0.02 mg/ml dexamethasone, and 0.16 mg/ml gentamicin (mLDG) for symptomatic support when esophagitis occurred. Patients in both groups stopped EGCG or mLDG solution two weeks after radiotherapy. This research design had been approved by our local study review board. All patients were included with written informed consent. **Figure 1** showed an overview of the study design. In the NCT02577393 study, there were 83 patients with lung cancer, including 38 patients with SCLC and 45 patients with non-small cell lung cancer. All 38 patients with SCLC were included in this observational non-interventional study and followed up. Follow-up visits with H&P and chest CT occurred every 3–4 months for the first two years, every 6 months for the following three years, and annually thereafter.

## Radiotherapy Details

Radiotherapy was administered through three-dimensional conformal or intensity-modulated techniques. All patients underwent CT simulation and were immobilized supinely on thermoplastic masks or vacuum molded bags. Gross target volume included post-chemotherapy primary tumor and pre-chemotherapy nodal volume. The total dose was 50.4–60 Gy (a fraction of 1.8–2Gy once a day) or 45 Gy (1.5 Gy twice a day) for five days weekly. Planning target volume was encompassed by the 95% isodose, and the maximum dose is below 107%. The dose limits for organ at risk were as before, such as less than 18 Gy mean lung dose (10).

### Study Assessments

The tumor (T), node (N), and metastasis (M) of SCLC were graded by the eighth edition AJCC/UICC stage classification. ARIE was assessed according to RTOG scoring criteria weekly from onset of radiation to 2 weeks after completion of radiation. Esophagitis-related pain and dysphagia were graded by the numerical rating scale. Esophagitis-related indexes (adjusted esophagitis index, AEI; adjusted pain index, API; adjusted dysphagia index, ADI) were calculated as previously reported (10) and shown in **Supplementary Figure 1**. The curve of each patient was drawn with the grade of esophagitis-related parameters (ARIE, pain, and dysphagia score) as ordinate and the observation completion rate as abscissa. The area under the three curves, namely AEI, API, or ADI, was an integrated measurement of severity and duration of esophagitis from different perspectives.

Tumor response was assessed by RECIST criteria. ORR included complete and partial response (CR and PR) rate. PFS referred to the time from randomization until tumor progression or death from any reasons or the last medical observation. OS encompassed the intervening time from randomization to death or the last medical observation.

In this study, we primarily assessed the differences between the two groups in terms of objective response rate (ORR) and progression-free survival (PFS). The secondary endpoints included OS and three adjusted esophagitis-related indexes. In order to avoid subjective deviation, an independent evaluation group composed of two senior and well-equipped doctors assessed the above endpoints without knowing the treatment allocation.

### Data Statistics

Updated data, covering the period until January 1, 2021, were used for this assessment. The calculation method of the sample size had been clarified in the previously released NCT02577393 research report, while the current analysis of ORR, PFS, and OS in SCLC was not informed. Kaplan–Meier curves and estimates were used to deal with differing survival data including PFS, OS and follow-up time. The 1-, 2-, and 5-years PFS rates were compared by Z test. Univariate and multivariate Cox regression analyses were applied to analyzed variables affecting survival time. The differences between categorical variates were tested by Fisher’s exact test. The measurement data of the different groups were analyzed by t-test. Statistical significance for the hypothesis was set at a *P*-value less than 0.05 with a two-sided version. The statistical analysis was carried out using statistical package for the social sciences software systems (v. 17.0).

## Result

### Baseline Characteristics

In this report, the first patient was enrolled in April 2015 and the last one in April 2018. Thirty-eight patients with SCLC were eligible for analysis. The differences in baseline characteristic variables between the two groups were not significant (**Table 1**). Eight (21.1%) of them were female. Their age was 41 to 75 years old (median 58 years). 44.7% of the patients undertook radiotherapy and chemotherapy at the same time. Dosimetry parameters predicting potential radiation toxicities for esophagus were described in detail: the mean and maximum values were 32.7 Gy (20.3–51.9) and 65.3 Gy (51.2–68.6); V30, V35, and V50 were 57.3, 54.5, and 42.0%, respectively.

**Table 1:** Pretreatment characteristics.

Characteristic	EGCG application (n = 26)	conventional treatment (n = 12)	*P*
Age (years)			
Median (range)	56.5 (41–75)	62.5 (50–70)	0.282
Sex (n)			
Male	22	8	0.23
Female	4	4	2
KPS score (n)			
80	10	3	0.48
90	16	9	6
Smoking index (years*root)			
Median (range)	400 (0–1,600)	500.00 (0–1,600)	0.790
T (n)			
1	0	1	0.17
2	5	3	7
3	12	2	
4	9	6	
N (n)			
1	1	0	0.185
2	11	2	
3	14	10	
Treatment (n)			
Sequential CRT	14	7	1.00
Concomitant CRT	12	5	0
Esophageal dosimetric parameters			
Mean value (Gy)			
Median (range)	31.0 (20.3–51.9)	34.2 (20.3–43.5)	0.505
Maximum value (Gy)			
Median (range)	65.8 (51.2–68.6)	64.9 (57.2–67.8)	0.711
V30 value (%)			
Median (range)	54.8 (27.0–80.0)	57.8 (30.0–72.0)	0.493
V35 value (%)			
Median (range)	52.0 (22.0–75.0)	55.0 (25.0–65.0)	0.449
V50 value (%)			
Median (range)	37.0 (19.0–70.0)	46.0 (10.0–58.0)	0.532

### Acute Esophagitis

**Figure 2:**
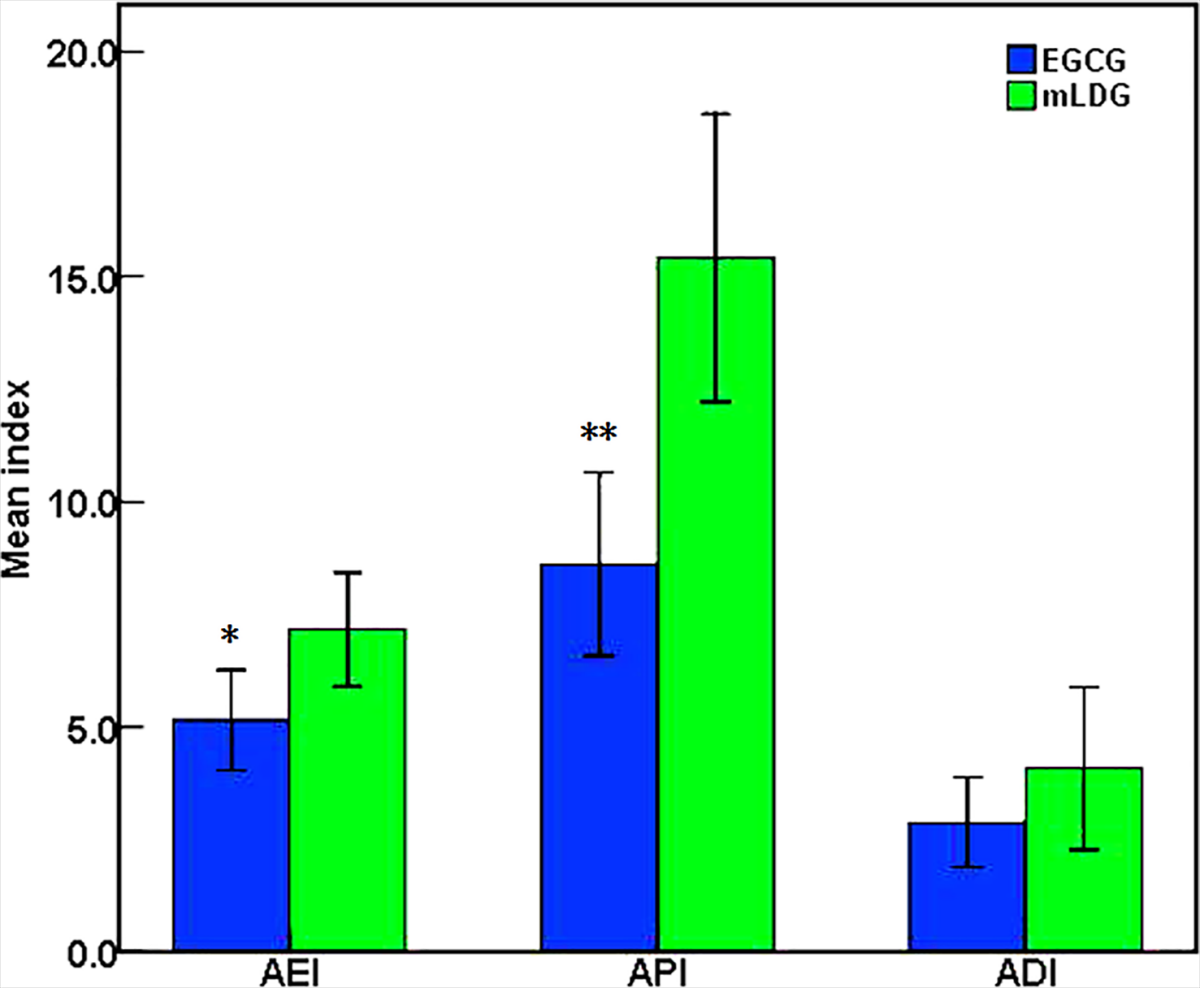
EGCG significantly improved the patient’s esophagitis and pain compared with conventional treatment. The statistical differences were observed in the mean value of AEI and API between EGCG and mLDG groups (*P = 0.030; **P < 0.001).

The median onset time of ARIE and pain symptom for patients was 3 weeks (range, 2–5 weeks) and that of dysphagia symptom was 3 weeks (range, 2–7 weeks). **Table 2** showed the highest grades of ARIE, pain, and dysphagia endured by patients in the two groups during treatment, and there were no statistical differences (*P* = 0.441; *P* = 0.796; *P* = 0.394). The mean AEI and API of patients with EGCG application were significantly lower than those of patients with mLDG mixture solution (5.15 ± 2.75 *vs* 7.17 ± 1.99, *P* = 0.030; 8.62 ± 5.04 *vs* 15.42 ± 5.04, *P* < 0.001 **Figure 2**). However, no statistical difference was observed in ADI (2.88 ± 2.47 *vs* 4.08 ± 2.84, *P* = 0.193; **Figure 2**). There was no significant difference in AEI, API, and ADI between patients receiving concurrent radio-chemotherapy and patients receiving sequential radio-chemotherapy in mLDG group and/or EGCG group (all *P* > 0.05).

**Table 2:** Distribution of maximum grade of esophagitis-related parameters and tumor response.

	EGCG application	conventional treatment	Total	*P*
Tumor response				
Complete response	3(11.5%)	1(8.3%)	4(10.5%)	
Partial response	19(73.1%)	5(41.7%)	24(63.2%)	
Stable disease	2(7.7%)	3(25.0%)	5(13.2%)	
Progressive disease	2(7.7%)	3(25.0%)	5(13.2%)	*P* = 0.145
Overall response	22(84.6%)	6(50%)	28(73.7%)	
Overall non-response	4(15.4%)	6(50%)	10(26.3%)	*P* = 0.045
Maximum acute radiation-induced esophagitis grade				
1	21(80.8%)	9(75.0%)	30(78.9%)	
2	5(19.2%)	2(16.7%)	7(18.4%)	
3	0(0%)	1(8.3%)	1(2.6%)	*P* = 0.441
Maximum pain grade				
1	1(3.8%)	0(0%)	1(2.6%)	
2	6(23.1%)	1(8.3%)	7(18.4%)	
3	15(57.7%)	8(66.7%)	23(60.5%)	
4	2(7.7%)	2(16.7%)	4(10.5%)	
5	1(3.8%)	0(0%)	1(2.6%)	
6	1(3.8%)	1(8.3%)	2(5.3%)	*P* = 0.796
Maximum dysphagia grade				
1	5(19.2%)	0(0%)	5(13.2%)	
2	18(69.2%)	10(83.3%)	28(73.7%)	
3	3(11.5%)	2(16.7%)	5(13.2%)	*P* = 0.394

### Response Rates to Cancer Therapy

Overall, radiographic remission was observed in 73.7% of patients after the end of tumor treatment. No significant difference was noted between the groups in terms of CR or PR separately (three of 26 in EGCG group *vs* one of 12 in the placebo group, *P* = 1.000; 19 of 26 in EGCG group *vs* five of 12 in the placebo group, *P* = 0.081). The ORR of patients with EGCG was slightly higher than that of patients with conventional therapy (*P* = 0.045, **Table 2**). In the univariate regression analysis, EGCG application was positively correlated with ORR (*t* =2.355, *P* = 0.024), and N stage was negatively correlated with ORR (*t* = −2.071, *P* = 0.046). In multivariate stepwise logistic regression analysis, only EGCG application was still significantly correlated with ORR.

### PFS and OS Analyses

At the deadline for data collection, the median follow-up was 56.0 months [95% confidence interval (CI): 37.1–74.9] for patients in the EGCG group. Whereas for patients in the mLDG group, it was 50.0 months (95% CI: 28.2–71.8). Twenty-three patients died, twenty from disease progression, two from heart failure, and one from radiation pneumonia. One patient in each group was lost to follow-up.

**Figure 3:**
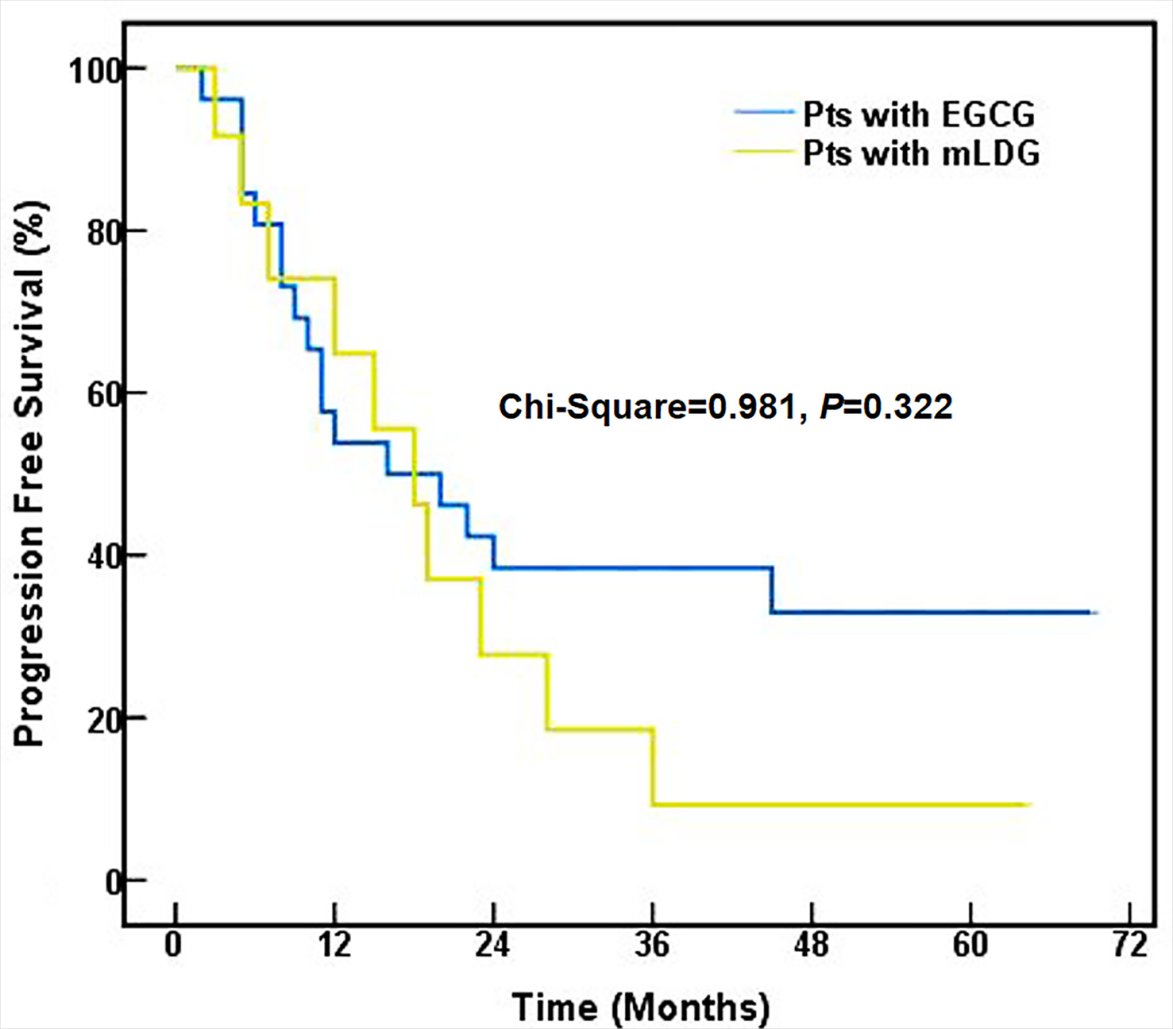
Progression-free survival of SCLC patients (pts) treated with EGCG (blue) or mLDG (gold) solution.

**Figure 4:**
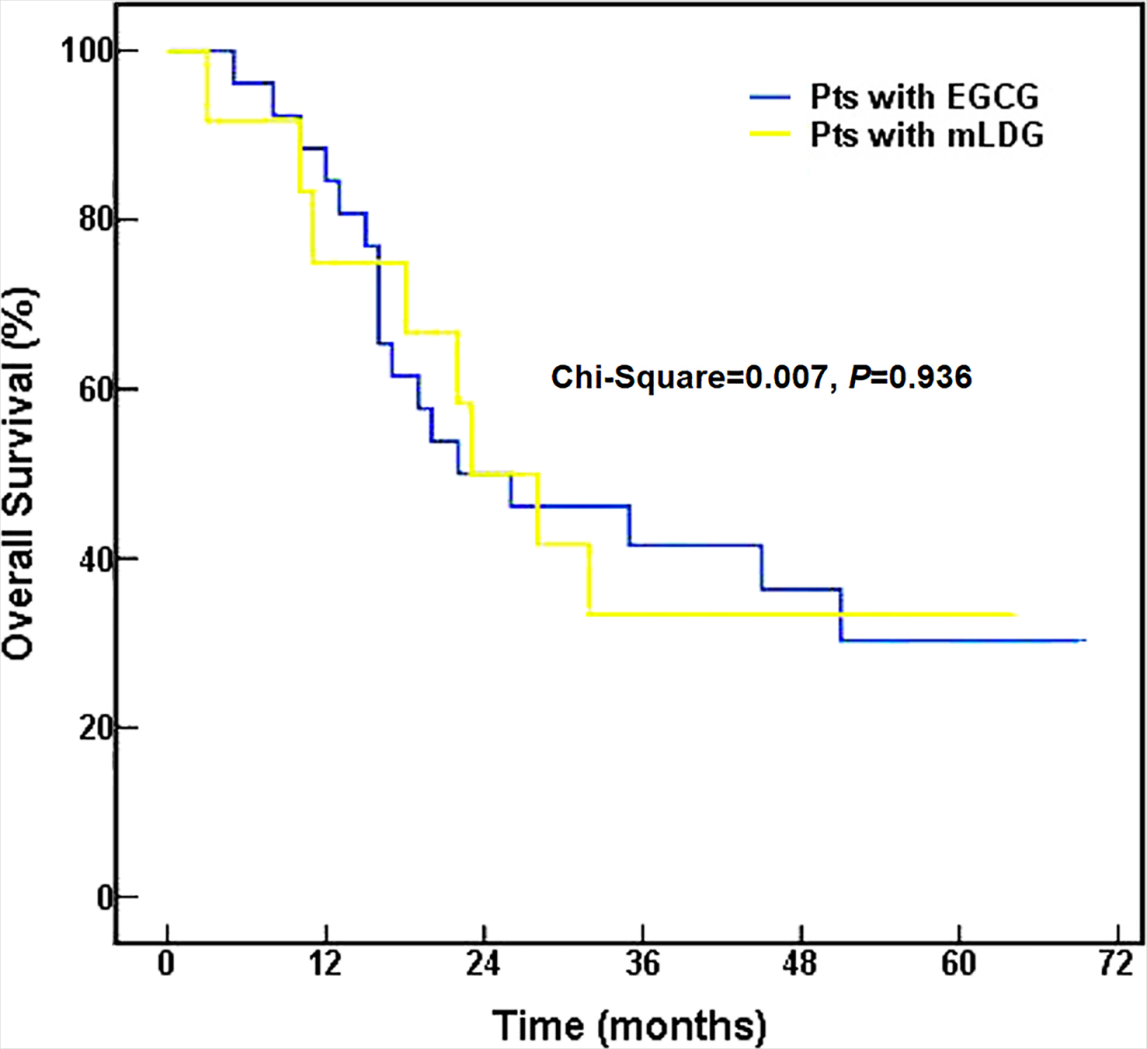
Overall survival of SCLC patients (pts) treated with EGCG (blue) or mLDG (gold) solution.

The median time to PFS was 16.0 months (95% CI, 2.3–29.7) for EGCG and 18.0 months (95% CI, 11.6–24.4) for mLDG. Mean (standard error) PFS time was 31.9 (5.5) months for EGCG and 21.2 (5.0) months for mLDG. There was no statistical difference in PFS between the two group (chi-Square = 0.981, *P* = 0.322, **Figure 3**). The 1-, 2- and 5-year PFS rates in patients with EGCG solution were 53.8, 38.5, and 33.0%, respectively, and those in patients with mLDG solution were 64.8, 27.8, and 9.3%, respectively. The differences in PFS rates from 1 to 5 years were also insignificant (P = 0.320; P = 0.257; P = 0.076). The median OS in the EGCG group was 22.0 months (95% CI: 3.0–41.0), and the OS at 1, 2, and 5 years was 84.6, 50.0, and 30.3%, respectively. While the median OS of patients with mLDG was 23.0 months (95% CI: 12.8–33.2), and the OS at 1, 2, and 5 years was 75.0, 50.0, and 33.3%, respectively. Mean (standard error) OS times for EGCG and mLDG were 36.2 (4.9) and 33.6 (6.6) months, respectively. There was also no statistical difference in OS between the two groups (chi-Square = 0.007, *P* = 0.936, **Figure 4**).

Baseline and on-treatment factors associated with survival were analyzed. The correlation was only observed between the ORR and PFS (*P* = 0.002; hazard ratio (HR): 3.7, 95% CI: 1.6 to 8.3). For all subsets of participants examined, those with ORR had the higher PFS rates (1 year: 66.9 *vs* 30%; 2 years: 44.6 *vs* 10%; 5 years: 35.8 *vs* 0%). Additionally, low smoking index was associated with a prolonged OS (P = 0.044; HR: 1.0, 95% CI: 1.0 to 1.1) in a Cox proportional hazards regression analysis. The 1-, 2-, and 5-year overall survival rates separately were 94.7, 68.4, and 51.0% for patients with smoking index less than 500.

### Updated Adverse Event Data

Most of the adverse events (AEs) were similar to previously published data, and the EGCG-related AE were expected (10). The most common adverse event in patients was leukopenia. No Grade >3 hematological adverse event was perceived including deficiency of hemoglobin, leukocyte, and platelet. In addition to hematological toxicity, other grade ≥3 AEs were gastrointestinal reactions (two cases) and radiation-induced pneumonitis (one case), which were considered to be induced by radiotherapy and chemotherapy. Weight loss of more than 5% was seen in 10.5% of individuals, and weight increase of more than 5% was seen in 7.9%. There was no significant difference in weight change between EGCG and mLDG groups. A low rate of late radiation-induced dysphagia of 5.3% was observed but without statistical difference between the two groups. All adverse reactions mentioned above should be absent from the EGCG or mLDG applications. Only one case experienced mild queasiness while swallowing the EGCG solution, which could be associated with EGCG and attributed to its weird uncomfortable taste. No other adverse effects of EGCG were noted.

## Discussion

Until now, the standard initial therapy remains concurrent chemoradiotherapy (CRT) in most limited stage SCLC cases (17, 18). ARIE, as a frequent acute complication of CRT, can continuously reduce the quality of life of patients. The advanced radiotherapy approaches cannot solve this problem completely (19, 20). Radioprotectants may shed new light on potential breakthroughs. The four key requirements for the development of radioprotectants are effective protection of normal tissue, low drug toxicity, convenience for clinical applicability, and no repair of radiation-damaged cancer (21). Due to the failure to meet all the above conditions, amifostine, the only radiation protective agent approved by FDA, is not widely used in clinical practice (22, 23). EGCG, as a representative bioactive ingredient from medicine food homology, gradually stands out among many potential new radioprotectants (24). The safe pharmacology spectrum of EGCG was determined at six escalated dose levels in our previous phase I study, resulting in a recommended concentration of 440 umol/L (16). A subsequent single-arm study was launched to ensure its efficacy in the treatment of ARIE (15). Our prospective controlled trial NCT02577393 confirmed that EGCG had significantly reduced esophagitis than conventional treatment (mLDG) during chemoradiotherapy for lung cancer, especially when used for prophylaxis (10). The above studies preliminarily proved that EGCG could meet the first three of the mentioned four key requirements. The minimum requirement for the application of protectors was not to reduce the anti-tumor effect of radiation, preferably to enhance it. This study was the first to report the long-term follow-up data of EGCG in patients with SCLC after chemoradiotherapy. To explore whether ECGC as a radioprotective agent for esophageal tissue would affect the short-term and long-term efficacy of radiotherapy for lung cancer, the differences of objective tumor remission rate and the follow-up survival time between EGCG group and placebo group were taken as the clinical endpoints (25–27).

The observations on EGCG efficacy in improving radiation-induced esophagitis were generally consistent with previous studies (10). Even if the number of participants was small, there was a significant difference in the AEI. The statistical difference was also found in the pain index. The significant difference was not found between the EGCG and mLDG groups with regard to ADI and the maximum grade of ARIE. Similar to previous studies, there was an increasing trend in the severity of esophagitis with concurrent CRT compared with sequential CRT (28). It did not reach statistical significance due to the small number of participants; therefore no attempt was made in the study to evaluate the effect of EGCG on the toxicity of concurrent chemotherapy. Our data showed a low rate of late radiation-induced dysphagia in both groups. No new adverse reactions related to EGCG had been found.

In terms of short-term response, we separately compared tumor CR and PR between patients with EGCG or mLDG solution, but found no statistical significance. However, the ORR (CR plus PR rate) was higher in patients receiving EGCG solution than that in patients undertaking the conventional treatment. It had also been reported that EGCG can improve the short-term efficacy of radiotherapy in patients with breast cancer (29). In the long-term follow-up, EGCG-treated group had a shorter median PFS but a longer mean PFS compared to the mLDG-treated group, and the difference was not statistically significant. The 1-year PFS of mLDG was higher than that of the EGCG group while the 2- and 5-year PFS rates show reversal. The 5-year PFS rate difference between EGCG and the control group was 27% (33 *vs* 9%). The undesirable performance of 1-year PFS in the EGCG group could be caused by non-cancer death and the insufficient number of participants. The association between EGCG application and ORR or between ORR and PFS appeared, but the association between EGCG application and PFS was not shown. The differences of OS between EGCG and mLDG groups also failed to reveal an obvious statistical difference. Though an overall statistically beneficial effect of EGCG was not found in the study, the trends suggested that it could bring a clinical benefit in SCLC patients with RT. EGCG remarkably enhanced the efficacy of tumor radiotherapy in the short-term and had a tendency to increase it in the long-term.

The mechanism of EGCG is complicated. Radiation essentially destroys the living organism by the deposition of energy directly into key biological macromolecules such as desoxyribonucleic acid (DNA), and a series of cascading reactions are triggered by the production of reactive oxygen species (30). EGCG can directly reduce radiation-induced DNA breaks and has the anti-ROS activity, anti-inflammatory response, anti-apoptosis function (31–33). EGCG also influences epigenetic changes through altering histone acetylation and DNA methylation (34–36). Surprisingly, it has been reported that EGCG can significantly reduce the damage of normal mouse liver cell lines induced by radiation and effectively increase the radiosensitivity of mouse liver cancer cells at the same time. EGCG combined with radiotherapy can further reduce the expression of the apoptosis suppressor bcl-2 and increase the expression of apoptosis-related proteins in hepatocellular carcinoma cell lines. However, the opposite effects are exerted on mouse liver cell line. The regulatory effect of EGCG may be attributed to the different expression of miR-34a in two cells (37). The researchers also discovered that miR-34 methylation in small cell lung cancer is lower than that in normal cells (38). Further *in vivo* and *in vitro* tests are needed to control the influencing factors, examine the conclusions, and explore possible mechanisms.

Based on the above discussion, EGCG is very suitable as a radioprotectant for patients with SCLC who undergo radiotherapy. Still, several weaknesses of the research should be pointed out. At first, the number of patients in the study is small. As a follow-up observational study on a new drug, the study here cannot continue to recruit more patients, and only patients from the NCT02577393 study are screened for analysis. Evaluating with fewer patients sometimes fails to reach statistical significance (39). For example, the 5-year PFS rate difference between the two groups was 27% with P >0.05. Moreover, the absence of stratified randomization by chemoradiotherapy scheme could also affect the accuracy of prognostic conclusions, though the clinical features of patients between EGCG and mLDG group are well balanced at baseline. Lastly, there is a lack of research on the intricate molecular mechanism underlying different effects of EGCG on tumor and esophageal tissue. Nonetheless, it may be stated that the results of the pilot study support further exploration of the EGCG application in patients with ARIE.

## Conclusion

Consistent with previous reports, EGCG could alleviate some esophagitis-related indexes in SCLC patients receiving radiotherapy with an acceptable toxicity. Furthermore, EGCG may increase the ORR without reducing PFS or OS. Further basic and clinical studies should be conducted to testify and clarify the mechanisms of differential effect of EGCG on cancer and normal tissues during radiation.

## Data Availability Statement

The datasets used and analyzed during the current study are available from the corresponding authors on reasonable request. Requests to access these datasets should be directed to HZ, zhx87520052@163.com.

## Ethics Statement

The studies involving human participants were reviewed and approved by the ethics committee of Shandong Cancer Hospital and Institute. The patients/participants provided their written informed consent to participate in this study.

## Author Contributions

HZ and JY contributed to the design of the research. WZ and YZ were involved in collecting data and drafting of the manuscript. XL, SZ, and LX planned the therapy. HZ, XL, LX, and SZ devoted to collecting information, analyzing data, and modifying content. All authors contributed to the article and approved the submitted version.

## Funding

This research was funded by National Natural Science Foundation of China (82003233), Shandong Provincial Natural Science Foundation (No. ZR2016HM35), Key Scientific and Technological Projects of Shandong Province (2018GSF118232), and Jinan Science and Technology Plan Project (202019163).

## Conflict of Interest

The authors declare that the research was conducted in the absence of any commercial or financial relationships that could be construed as a potential conflict of interest.

## Supplementary Material

Supplementary Figure 1 Calculation methods of adjusted ARIE-related indexes. The trapezoid formula was used to calculate the area beneath the curve graph of each patient. Taking the AEI of a case in the figure as an example, when the horizontal axis was the percentage of observation completion and the vertical axis was the grade of ARIE, the calculated value was 14.
