# Supplementary figures and images for: Evaluation of Epigallocatechin-3-Gallate as a Radioprotective Agent During Radiotherapy of Lung Cancer Patients: A 5-Year Survival Analysis of a Phase 2 Study
Source: Front Oncol. 2021 Jun 10;11:686950. doi: 10.3389/fonc.2021.686950 (PMC8223749; doi:10.3389/fonc.2021.686950)

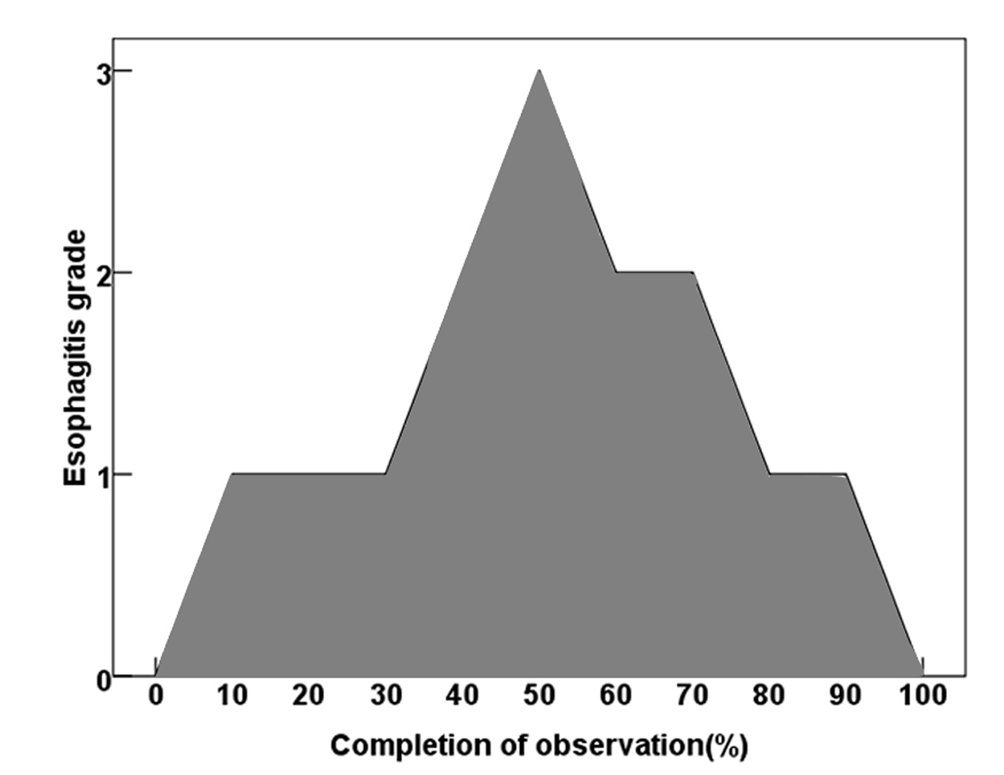

Supplement: Supplementary Figure 1 — Calculation methods of adjusted ARIE-related indexes. The trapezoid formula was used to calculate the area beneath the curve graph of each patient. Taking the AEI of a case in the figure as an example, when the horizontal axis was the percentage of observation completion and the vertical axis was the grade of ARIE, the calculated value was 14. [file Image_1.tif]
