# Supplementary material for: Evaluation of Epigallocatechin-3-Gallate as a Radioprotective Agent During Radiotherapy of Lung Cancer Patients: A 5-Year Survival Analysis of a Phase 2 Study
Source: Front Oncol. 2021 Jun 10;11:686950. doi: 10.3389/fonc.2021.686950 (PMC8223749; doi:10.3389/fonc.2021.686950)
Supplement: Supplementary file 2 [file Table_1.doc]

Table S1. RTOG acute radiation-induced esophagitis scoring criteria

| 0 | I | II | III | IV |
| --- | --- | --- | --- | --- |
| No change over baseline | Mild dysphagia or odynophagia/may require topical anesthetic or non-nacrotic analgesics/may require soft diet | Moderate dysphagia or odynophagia/may require narcotic analgesics/may require puree or liquid diet | Severe dysphagia or odynophagia with dehydration or weight loss>15% from pretreatment baseline requiring N-G feeding tube, i.v. Fluids or hyperalimentation | Complete obstruction, ulceration, perforation,fistula |
